# The Role of Endogenous Eicosapentaenoic Acid and Docosahexaenoic Acid-Derived Resolvins in Systemic Sclerosis

**DOI:** 10.3389/fimmu.2020.01249

**Published:** 2020-06-19

**Authors:** Aslıhan Avanoǧlu Güler, Francesca Wanda Rossi, Silvia Bellando-Randone, Nella Prevete, Abdurrahman Tufan, Mirko Manetti, Amato de Paulis, Marco Matucci-Cerinic

**Affiliations:** ^1^Department of Experimental and Clinical Medicine, University of Florence and Department of Geriatric Medicine, Division of Rheumatology AOUC, Florence, Italy; ^2^Department of Internal Medicine, Division of Rheumatology, Gazi University Faculty of Medicine, Ankara, Turkey; ^3^Department of Internal Medicine, Clinical Immunology and Rheumatology, University of Naples Federico II, Naples, Italy

**Keywords:** resolvins, resolution of inflammation, systemic sclerosis, innate immunity, adaptive immunity, fibrosis

## Abstract

Resolvins, the member of specialized pro-resolving mediators, are produced from omega-3 polyunsaturated fatty acids as a response to an acute inflammatory process in that termination and resolution of inflammation. In the acute inflammation, these lipid mediators limit polymorphonuclear cells infiltration, proinflammatory cytokine production; promote efferocytosis, and regulate several cell types being important roles in innate and adaptive immunity. Any dysregulation or defect of the resolution phase result in prolonged, persistent inflammation and eventually fibrosis. Resolvins are implicated in the development of various chronic autoimmune diseases. Systemic sclerosis (SSc) is a very complicated, chronic autoimmune disorder proceeding with vasculopathy, inflammation, and fibrosis. Dysregulation of innate and adaptive immunity is another important contributing factor in the pathogenesis of SSc. In this review, we will focus on the different roles of this new family of lipid mediators, characterized by the ability to prevent the spread of inflammation and its chronicity in various ways and how they can control the development of fibrotic diseases like SSc.

Systemic sclerosis (SSc) is a complex immune-mediated connective tissue disorder characterized by microvascular damage, inflammatory cell infiltration, and excessive deposition of extracellular matrix proteins (ECMs) in the skin and various internal organs (1–3). Over the course of the disease, these pathologic alterations cause severe organ dysfunctions such as pulmonary fibrosis, pulmonary arterial hypertension, cardiac arrhythmias and heart failure, which are the major causes of mortality in SSc (4). Identification of the immune, vasculopathic, and fibrotic mechanisms involved in the pathogenesis of SSc is critical for the understanding of disease progression and developing new disease-modifying therapies (5). Despite the fact that innate and adaptive immunity components, including T cells, B cells, macrophages, dendritic cells (DCs), and multiple cytokines (e.g., interleukin (IL)-4, IL-6, transforming growth factor (TGF)-β) play roles in both the onset and the progression of SSc, the exact etiopathogenesis of the disease still remains elusive (1, 2).

It is well-known that acute inflammatory responses, triggered by a variety of noxious stimuli, including endogenous and exogenous signals, are protective, self-limited reactions that are essential for restoring homeostasis in the affected tissues. However, this benign process may not subside, leading to chronic systemic inflammatory disorders (6, 7). In fact, in a few autoimmune and chronic inflammatory diseases, including SSc, perturbation is observed in inflammation resolution (8). Until recently, termination of acute inflammation was considered as a passive process. However, the latest investigations have demonstrated that the resolution of inflammation is an active process controlled by the local release of various mediators called specialized pro-resolving mediators (SPMs). The biosynthesis of SPMs is induced by pro-inflammatory stimuli as a compensatory mechanism to downregulate the inflammatory response (7). In general, SPMs bind to G protein-coupled receptors (GPRs) to exert anti-inflammatory and pro-resolving biological actions; inhibition of polymorphonuclear leukocyte (PMNs) infiltration and pro-inflammatory cytokine/mediator secretion; promote bacterial removal; and evoke the efferocytosis of apoptotic PMNs through macrophages (9, 10). Thus, far, more than 20 different SPMs have been explored using lipid mediator metabolon lipidomics and proteomics, and these SPMs have been subdivided into four main classes based on distinct biosynthetic pathways and target receptors: lipoxins, resolvins (Rvs), protectins, and maresins (11, 12). The discovery of Rvs attracted significant interest because these lipid mediators play prominent roles in different pathological conditions by sustaining homeostasis owing to their anti-inflammatory properties (13). It is widely accepted that Rvs play significant roles in several chronic inflammatory diseases, such as rheumatoid arthritis, Sjogren's syndrome, and inflammatory bowel disease (14–17). Although many experimental studies have been conducted to define the preventive role of Rvs in pulmonary fibrosis and ischemia-reperfusion injury in animal models, their contribution to the pathogenesis of SSc is yet to be clarified (18, 19). In this review, we will focus on this new family of lipid mediators that can control the propagation and prolongation of inflammation, as well as their possible roles in the pathogenesis of fibrotic diseases such as SSc.

## Biosynthesis of Resolvins and Their Receptors

For the first time, Rvs were identified in inflammatory exudates triggered by tumor necrosis alpha (TNF-α) in mice exposed to omega (Ω)-3 polyunsaturated fatty acids (PUFAs) and aspirin. Usually, different immune cells, such as macrophages and PMNs generate Rvs from Ω-3 PUFAs, namely docosahexaenoic acid (DHA) and eicosapentaenoic acid (EPA), originating from the dietary sources and cell membranes through phospholipase enzyme pathways (20). Two classes of Rvs have been identified: D-series resolvins (RvD1-6) derived from DHA through lipoxygenase (LO)-initiated pathways during the inflammation- resolution phase and the E-series family of Rvs (RvE1-4) produced from EPA via 5-LO and cytochrome P450 (12, 21–23). It has been shown that Rvs signal through specific GPRs (23–26).

RvD1 exerts anti-inflammatory and inflammation-resolution effects via A lipoxin and formyl peptide receptor 2 (ALX/FPR2), as well as via GPR32 (24, 25). RvD2 interacts with GPR18 expressed on PMNs, monocytes, and macrophages (26). In addition to RvD1, RvD3, and RvD5 activate GPR32 and enhance the phagocytosis and inhibition of neutrophil transmigration (27, 28). Furthermore, RvEs exert their function through GPCRs. Chemerin receptor 23 (ChemR23), which is selectively expressed on antigen-presenting cells (APCs), is a binding site for RvE1 (29). Additionally, RvE1 also interacts with leukotriene B_4_ receptor 1 (BLT1), as a partial agonist, which mediates the potent inflammatory effects of leukotriene B4 (LTB_4_) (30).

## Role of Resolvins in the Innate Immunity

**Figure 1:**
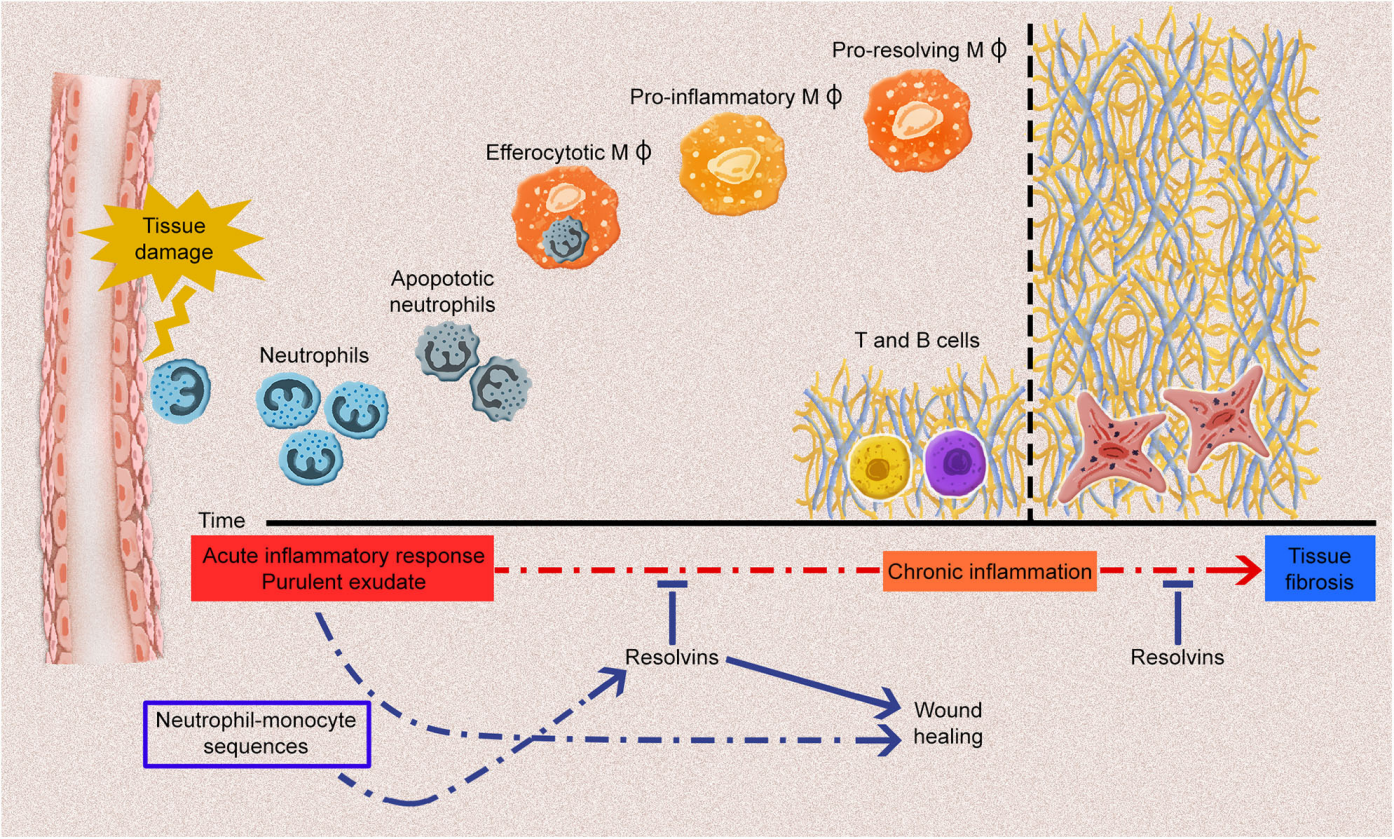
The acute inflammatory response and potential fates for the acute inflammatory process. Tissue damage induced by endogenous or exogenous stimuli leads to the generation of acute inflammatory responses, including various types of proinflammatory cell infiltrations and the production of plenty of proinflammatory mediators. Polymorphonuclear leukocytes infiltration especially neutrophils induce the influx of monocyte-derived macrophages to remove apoptotic cells and debris. Throughout the resolution phase of inflammation, resolvins (Rvs) promote the efferocytosis of macrophages and differentiation of proinflammatory macrophages (MΦ) into anti-inflammatory macrophages. At the post-resolution phase of inflammation, adaptive immunity response (B and T cells) establishes which contributes the wound healing. Any dysregulation of these processes may lead to chronic inflammation and fibrosis. Rvs limit the acute inflammatory process, thus, they prevent the development of chronic inflammation and fibrosis.

In SSc, endothelial cell activation is one of the earliest events, along with immune cell activation and inflammation (31). Unresolved or prolonged inflammation and immune cell activation could result in chronic inflammation and, subsequently, in fibrosis (7). The inflammatory process is characterized by the production of various mediators (e.g., cytokines, chemokines, vasoactive amines, and eicosanoids) by the innate immune cells, including PMNs, macrophages, dendritic cells, lymphocytes, endothelial cells, and fibroblasts, in the damaged tissue. This occurs concomitantly with the upregulation of cell-adhesion molecules on leukocytes and endothelial cells, and continue with the infiltration of neutrophils, monocytes, and phagocytes. It is now recognized that Rvs, which are produced by immune cells such as macrophages and PMNs, are pivotal in the resolution of acute inflammatory reactions. They significantly limit acute inflammation response and promote tissue repair (32) (Figure 1). Each type of Rvs is considered to have a unique role in the resolution phase of inflammation (Table 1). Therefore, the role of Rvs and their possible failure to resolve local inflammatory responses should be investigated in the pathogenesis of SSc.

**Table 1:** Resolvins and their functions on immune cells.

**Immune cells**	**Resolvin D-series**					**Resolvin E-series**				
	**RvD1**	**RvD2**	**RvD3**	**RvD4**	**RvD5**	**RvE1**	**RvE2**	**RvE3**	**RvE4**	**References**
PMNs migration infiltration	↓	↓	↓	↓	↓	↓	↓	↓	↓	(23, 26, 28, 30, 33–39)
Mediators	↑IL-10 ↓LTB_4_	↓IL-1β ↓IL-6 ↓IL-17 ↓IL-23 ↓TNFα ↓LTB_4_ ↓↑IL10	↓IL-6 ↓LTB_4_ ↑IL-10			↓IL-1β↓IL-6↓TNFα	↑IL-10	↓IL-4↓IL-5↓IL-13↓IL-23		(24, 26, 28, 34, 35, 37, 40–44)
Mφ efferocytosis	↑	↑	↑	↑	↑	↑	↑		↑	(23, 28, 34, 37, 38, 41, 45)
Mφ polarization	↑M2	↑M2								(46–49)
DCs						↓Migration ↓IL-12 ↓IL-23	↓IL-23	↓IL-23		(29, 35)
T cells	↓CD4^+^T ↓Th1 ↓Th17 ↓IFN-ɤ ↓IL-17 ↓CD8^+^T ↑Treg	↓CD4^+^T↓Th1↓Th17↓IFN-ɤ ↓IL-17↓CD8^+^T↑Treg				↓CD4^+^T ↓ IL-4 ↓IFN-ɤ ↓CD8^+^T				(44, 50, 51)
B cells	↑IgM ↑IgG ↓IgE									(52–54)

*DCs, dendritic cells; IL, interleukin; Ig, immunoglobulin; IFN-ɤ, interferon-gamma; LTB_4_, leukotriene B_4_; Mφ, macrophages; PMNs, polymorphonuclear leukocytes; RvD, resolvin D; RvE, resolvin E; Th, T helper; TNF-α tumor necrosis factor alpha; Treg, T regulatory cell*.

### Effect of Resolvin E-series on Inflammation

RvE1 inhibits human neutrophil transendothelial migration and infiltration *in vivo* (20). RvE1 is characterized by the modulation of leukocytes adhesion molecules through the enhancement of L-selectin shedding, which inhibits the aggregation of leukocytes and reduces CD18 (LFA-1) expression, which is required for neutrophils adhesion and transmigration (30, 33). Animal studies have elucidated that RvE1 enhances efferocytosis through macrophages and reduces pro-inflammatory cytokines, including IL-1β, IL-6, and TNF-α, in zymosan-induced peritonitis (40, 41). Similarly, the result of a mice animal model study indicated that the exogenous RvE1 induces the phagocytosis of neutrophil apoptosis via macrophages in pulmonary inflammation (45). Similar to RvE1, RvE2 actively participates in the resolution of inflammation by blocking neutrophil infiltration through chemotaxis modulation, reinforcement of phagocytosis, and macrophages-dependent production of IL-10 (34). Eosinophils mainly release RvE3, which limits the infiltration of PMNs in zymosan triggered peritonitis (55). In an allergic lung inflammation model, RvE3 significantly reduced the number of inflammatory cells and the secretion of pro-inflammatory cytokines in bronchoalveolar lavage (35). Recently, it has been demonstrated that the production of new RvE4 is accelerated by hypoxia, which induces the efferocytosis of neutrophils and erythrocytes through macrophages and inhibits the infiltration of neutrophil in hemorrhagic exudates *in vivo* (23).

### Effect of Resolvin D-series on Inflammation

RvD1 modulates the regulatory action of PMNs by inhibiting rolling and adhesion to endothelium via GPR32, in addition to limiting the infiltration of leukocytes and neutrophils via FPR2/ALX and the production of pro-inflammatory mediators in zymosan-induced peritonitis (36). Through this binding with FPR2/ALX, RvD1 inhibits lipopolysaccharide (LPS)-induced acute lung inflammation. This is realized because of reduced neutrophil infiltration due to the suppression of macrophage inflammatory protein (MIP)2-α (CXCL2) expression on alveolar macrophages (56). Similarly, RvD2 plays an effective role in the resolution phase of inflammation by reducing neutrophil recruitment, increasing mononuclear and macrophage phagocytosis by binding with GPR18, and suppressing the pro-inflammatory mediators (26). Additionally, RvD2 suppresses pro-inflammatory mediators by decreasing the plasma levels of IL-1β, IL-6, IL-17, IL-23, and TNF-α, as well as the levels of prostaglandin (PG)E2 and LTB_4_ in peritoneal exudates, as demonstrated using an animal sepsis model Interestingly, RvD2 decreases the plasma levels of the potent anti-inflammatory cytokine IL-10, which is of interest because of its detrimental impact on survival in sepsis (42). By contrast, RvD2 increases the level of IL-10 mRNA in the porphyromonas gingivalis-induced periodontitis (43). RvD3, which appears later than RvD1 and RvD2 in the resolution phase of inflammation, has potent local and systemic anti-inflammatory activities, such as decreasing the recruitment of PMNs and reducing the levels of IL-6 and LTB_4_ and matrix-degrading enzymes (MMP-2 and MMP-9). This enhances the level of IL-10 and stimulates macrophage efferocytosis (28, 37). Moreover, RvD4 decreases PMNs infiltration and promotes macrophage efferocytosis in zymosan-induced peritonitis and *Staphylococcus aureus*-triggered skin infection, in addition to inducing the phagocytosis of dermal fibroblasts (38). Several studies have elucidated the dysregulation of neutrophils in SSc and the relationship between neutrophil infiltration in lung tissue and lung fibrosis or disease severity (57–61). Considering all of these results, it seems that blocking of neutrophil migration and infiltration from most of Rvs might be beneficial for SSc or dysregulation of these mediators might contribute to the pathogenesis of SSc. As mentioned above, most of Rvs stimulate macrophage efferocytosis, which has been found to be dysfunctional in autoimmune diseases (systemic lupus erythematosus, Sjogren's syndrome, and SSc) (62–64).

### Polarization of Macrophages

From the onset of inflammation to its resolution, macrophages, as a part of innate immunity, play a significant role in inflammatory responses because they have possessed a diversity of phenotypes and polarization abilities. Based on responses to various signals from the environment, macrophages convert into classically activated M1 or alternatively activated M2 phenotypes that are mainly stimulated by interferon (IFN)-ɤ/LPS and IL-4/IL-13, respectively (65, 66). M1 macrophages contribute to the initiation and progression of inflammation by secreting pro-inflammatory mediators (IL-12, IL-1β, IL-6, and TNF-α). M2 macrophages, by contrast, are implicated in the tissue repair, wound healing, and resolution phase of the inflammation through the production of cytokines (IL-4, IL-10, IL-13) and growth factors (TGF-β, vascular endothelial growth factor (VEGF), and endothelial growth factor (EGF) (67). Alternatively activated M2 macrophages may have four subtypes: M2a stimulated by IL-4 or IL-13, M2b stimulated by immune complex and LPS, M2c stimulated by IL-10 and TGF-β1, and M2d stimulated by IL-6 and adenosine (68). Activated macrophages may change their polarization in accordance with new environmental stimuli (69). In autoimmune diseases, an M1/M2 imbalance has been detected. In SSc, M2 macrophages produce profibrotic cytokines that promote ECM synthesis (31). The M2 polarization observed in SSc seems to be induced by increased IL-6 and IL-4 levels (2). Although previous results are mainly consistent with M2 activation, recent evidence has suggested that macrophages express mixed surface markers of the M1 and M2 phenotypes in SSc (70–72).

During the resolution phase of inflammation, M1 macrophages change into the M2 phenotype owing to the action of specific mediators, especially SPMs. It has been shown that RvD1 significantly reduces the expression of M1 phenotype markers (TNF-α, IL-6, monocyte chemoattractant protein (MCP)-1 expression) and increases the expression of M2 markers in peritoneal macrophages obtained from obese mice (46). In the mouse carotid ligation model, systemic RvD2 markedly enhanced the proportion of M2 macrophages among the monocytes/macrophages present in the injured arterial wall (47). Recently, an assessment of inflammation in an abdominal aortic aneurysm model animal study demonstrated that RvD2 improved M2 polarization and ameliorated pro-inflammatory markers (IL-1β, IL-6, MCP-1, and MIP-1α) (73). Moreover, RvD1 reinforced the activation of M2 macrophages in acute smoke-induced lung inflammation (48). The animal study has indicated that long-term treatment with aspirin-triggered (AT) RvD1 does not influence macrophage polarization in long-term smoke-induced lung inflammation. Additionally, tissue fibrosis is not observed with long-term AT-RvD1 treatment (49). These effects could suggest that the effects of RvD1 on macrophage polarization may be associated with the type of inflammation (acute or chronic) or the duration of Rvs exposure. At the moment, we don't have enough data to disclosure if M2 differentiation may be a negative event in the pathogenic cascade of SSc linked to the activity of Rvs. Therefore, it is still very difficult to regard M2 differentiation as in those by Rvs as beneficial or pathogenic.

### Dendritic Cells

Dendritic cells (DCs) are an important component of innate immunity. They recognize and present damage-associated molecular patterns and pathogens, as well as induce the adaptive immune response. Usually, DCs are composed of two main cell types: conventional (cDC) and plasmacytoid (pDC), which, especially, secretes interferon-alpha (IFN-α). Recent studies have revealed that pDCs infiltrate the skin and the lungs of SSc patients, and contribute to fibrosis and that the number of pDCs in the lungs of SSc patients correlates with the severity of the lung disease (74, 75). ChemR23, a receptor of RvEs, is highly expressed in pDCs, and it mediates the migration of pDCs to inflammatory sites (76, 77). In an animal study, ChemR23 deficiency in knockout mice reduced the migration of pDCs to atherosclerotic lesions (78). Only RvE1 restrained the migration of DCs and inhibited their production of IL-12 via ChemR23 (29, 79). The serum level of IL-12 is increased in patients with SSc (79). Although the role of cDCs in SSc is not as known as pDCs, the increase in the production of proinflammatory cytokines from cDCs is demonstrated in SSc (80).

## Role of Resolvins in Adaptive Immunity

### T Cells

Several reports have suggested that T cells, particularly CD4^+^ T helper 2 (Th2), play a significant role in both the inflammatory and fibrotic processes of SSc (81). Activated CD4^+^ Th2 cells produce the predominantly potent profibrotic cytokines IL-4 and IL-13, which induce fibroblast proliferation, their differentiation into myofibroblasts, and polarization of M2 macrophages. All of these features are implicated in the pathogenesis of SSc (2, 82, 83). Furthermore, IL-13 producing CD8^+^ T cells have been detected in the skin in the early phases of SSc (84). Studies have highlighted the importance of Rvs in T cell regulation. Exogenous RvD1 diminishes the infiltration of CD4^+^ and CD8^+^ T lymphocytes in endotoxin-induced uveitis (50). Similarly, in an animal study, it was found that exogenous RvE1 suppressed the infiltration of CD4^+^ and CD8^+^ T cells in atopic dermatitis in a dose-dependent manner. In addition, RvE1 treatment reduced the IL-4 and IFN-ɤ production of activated CD4^+^ T cells (51). Abnormal Th17 cell responses are encountered in many chronic inflammatory and autoimmune diseases (85). Th17 and IL-17 may play an important role in SSc due to proinflammatory and profibrotic effects. Some evidence has demonstrated that the level of Th17 and IL-17 increased in SSc (86–88). However, the results of several studies have not found an increase in the level of IL-17 in SSc. Therefore, the role of Th17 and IL-17 have not completely understood in the pathogenesis of SSc yet (89–91). RvD1 and RvD2 abate the inflammatory responses of activated CD8^+^ T, Th1, and Th17 cells by decreasing the production of TNF-α, IFN-ɤ, IL-2, and IL-17. RvD1 and RvD2 inhibit the differentiation of naïve CD4^+^ T cells into Th1 and Th17 cells while they improve the differentiation of CD4^+^ T cells into regulatory T (Treg) cells. However, they do not exert any effect on the apoptosis of both CD8^+^ and CD4^+^ cells (44). In contrast to RvD1-2, RvE1 amplifies the T cell apoptotic activity of DCs through indolamine 2,3 dioxygenase induction (92). Although the effect of RvE1 on Th17 is undefined, RvE1 diminishes the production of IL-23 and IL-6, which are crucial for the survival of Th17 cell, in allergic lung inflammation (93). Recently, it has been demonstrated that RvE1, RvE2, and RvE3 decrease the production of IL-23 from bone marrow DCs *in vitro*. In particular, the treatment of house dust-mite-sensitized mice with RvE3 promoted the reduction of inflammatory cells, including eosinophils, and decreased IL-23 and IL-17 levels in lavage fluid, thus supporting the role of RvE3 in the resolution of allergic airway inflammation (35). These effects of Rvs on T cell regulation might create a protective mechanism against the dysregulation of T cells in SSc.

### B Cells

In recent studies on SSc, the role of the B cells in the generation of fibrosis has been highlighted, especially in the lungs and the gastrointestinal tract (94, 95). In fact, an increase in the naive B cell count and a decrease in memory and regulatory B cell counts has been found in SSc. These impairments of B cell homeostasis result in the decline in the production of potent anti-inflammatory and anti-fibrotic cytokines (i.e., IL-10) and the enhancement of production of proinflammatory cytokines (i.e., IL-6) (96). However, information on the effects of Rvs on B cells is scarce. In the mouse spleen, 17-hydroxydosahexaenoic acid (17-HDHA) (a biomarker of Rvs), RvD1, and RvE1, but not RvD2 and RvD5, have been detected (52, 53). In activated B cells, RvD1 elevates antibody production, notably immunoglobulin (Ig)M, while 17-HDHA increases both IgM and IgG in proportion to the increasing differentiation of B cells into the antibody-secreting B cell phenotype (52). Interestingly, it has been found that RvD1 and 17-HDHA suppress the differentiation of naïve B cells into IgE-secreting cells, which induce a specific block of the epsilon germline transcription (54).

## Effect of Resolvins on Ischemia-Reperfusion-Induced Inflammation

Raynaud's phenomenon (RP) is frequently encountered in SSc, and it influences the acral blood flow (97). In primary RP, impaired arterial inflow induced by sympathetic vasoconstriction causes mild reversible microvascular sufferance. In SSc, the impairment of arteriolar inflow is not compensated for by endothelial-dependent vasodilation. In fact, the disease affects the endothelial cells that are injured or dysfunctional (98, 99). Therefore, prolonged vasoconstriction leads to a loss of endothelial junctions, enhanced inflammatory immune cell migration and infiltration, and microvessel permeability (100). Moreover, repeated and sustained vasoconstriction attacks result in ischemia-reperfusion (IR) injury, which promotes the production of various proinflammatory mediators and reactive oxygen species, activation and migration of PMNs, and interaction with endothelial cells. This causes further microvascular damage (101, 102).

In this context, based on the data available about Rvs role in IR-induced injury models, they can be considered operative in a condition such as RP in SSc as well. In the IR-induced model, the levels of endogenous DHA and all types of RvDs, apart from RvD1 and RvD3, are known to increase in the plasma, while only DHA, RvD1, and RvD3 are detected in the affected kidney tissue. Exogenous Rvs (composed of RvD1-3) limit the infiltration of PMNs, and the deposition of interstitial collagen. RvD1 has a protective capacity for the kidney when administered after the development of IR (39). It has been shown that RvD1 treatment protects the lung tissue from IR-induced inflammation, thus limiting the homing of inflammatory cells, production of proinflammatory mediators, and apoptosis (19). IR injury elicits mitochondrial dysfunction and augments excessive ROS production (103). RvD1 limits IR-triggered liver damage by reducing mitochondrial oxidative stress and regulating mitochondrial homeostasis (104). Furthermore, RvD2 diminishes the infiltration of PMNs through GPR18 in IR-induced lung injury (26).

## Role of Resolvins in Fibrosis

Fibrosis is the main eventual hallmark of SSc. Vasculopathy; immune dysregulation, including innate and adaptive immunity; and several cytokines contribute to the process leading to fibrosis. However, the exact mechanisms of fibrosis in SSc still remain undefined.

Rvs are mainly known for their anti-inflammatory and pro-resolutive effects. They prevent fibrosis by limiting inflammation, supporting efferocytosis, and suppressing proinflammatory and profibrotic cytokines. Furthermore, Rvs have direct anti-fibrotic effects: RvE1 prevents hepatic fibrosis induced by Schistosoma japonicum infection by decreasing the levels of fibrotic markers such as laminin, hyaluronic acid, procollagen type III, and type IV collagen (105). In the animal model study, the anti-fibrotic effects of RvE1 were evaluated by inducing unilateral ureteric obstruction. The interstitial fibrosis obtained using this model was driven not by an inflammatory process but by an irreversible surgical insult, and it was characterized by collagen deposition and the proliferation of α-smooth muscle actin (SMA)^+^ myofibroblasts. RvE1 treatment dramatically attenuated the accumulation of α-SMA^+^ myofibroblasts, deposition of type IV collagen, and production of platelet-derived growth factor (PDGF)-BB, which is a potent inducer of fibroblast proliferation through activation of the AKT and ERK pathways. Moreover, RvD1 markedly reduced myofibroblast accumulation, and mRNA levels of type I and III collagens in an injured kidney (106). In bleomycin-induced lung tissue, treatment with 17(R)-RvD1, an epimer of RvD1, diminished the mRNA-expression of IL-1β, TGF-β1, and connective tissue growth factor, in addition to sharply reducing the numbers of macrophages and neutrophils in the bronchoalveolar fluid. The anti-fibrotic capacity of 17(R)-RvD1 has been confirmed based on reductions in hydroxyproline content (marker of collagen deposition), type I collagen mRNA expression, and score of the fibrotic changes (via Ashcroft scale) in the lung tissue. Moreover, 17(R)-RvD1 treatment has anti-inflammatory and anti-fibrotic effects even when it is administered in the established fibrotic stage in lung tissue (18). Besides, RvD1 alleviates collagen deposition in heart tissue after a myocardial infarction (107).

Epithelial-mesenchymal transition (EMT) is thought to be a crucial mechanism in the development of fibrosis, particular in the lungs and kidneys (97, 98). In general, EMT is closely related to embryonic development, tissue repair, wound healing, and cell migration. During tissue repair or wound healing, epithelial cells lose their phenotype and gain mesenchymal phenotypes to produce fibroblasts and myofibroblasts (108). EMT may be a part of the cellular origins of fibrosis in SSc (109). The potent pro-resolving activity of RvD1 has been further investigated in a model of acute respiratory distress syndrome (ARDS) in which it was demonstrated that RvD1 can prevent EMT of lung epithelial cells with reversal of the TGF-β-smad2/3 signaling pathway and lung fibrosis via the FPR2/ALX receptor (110). Endothelial-to-mesenchymal (EndoMT) transition is also thought to play an important role in both SSc-related fibrosis and vasculopathy (101, 102). Of note, RvD1 has also been reported to significantly inhibit TGF-β1-induced EndoMT through increasing the expression of Smad7 (39).

## Conclusion

**Figure 2:**
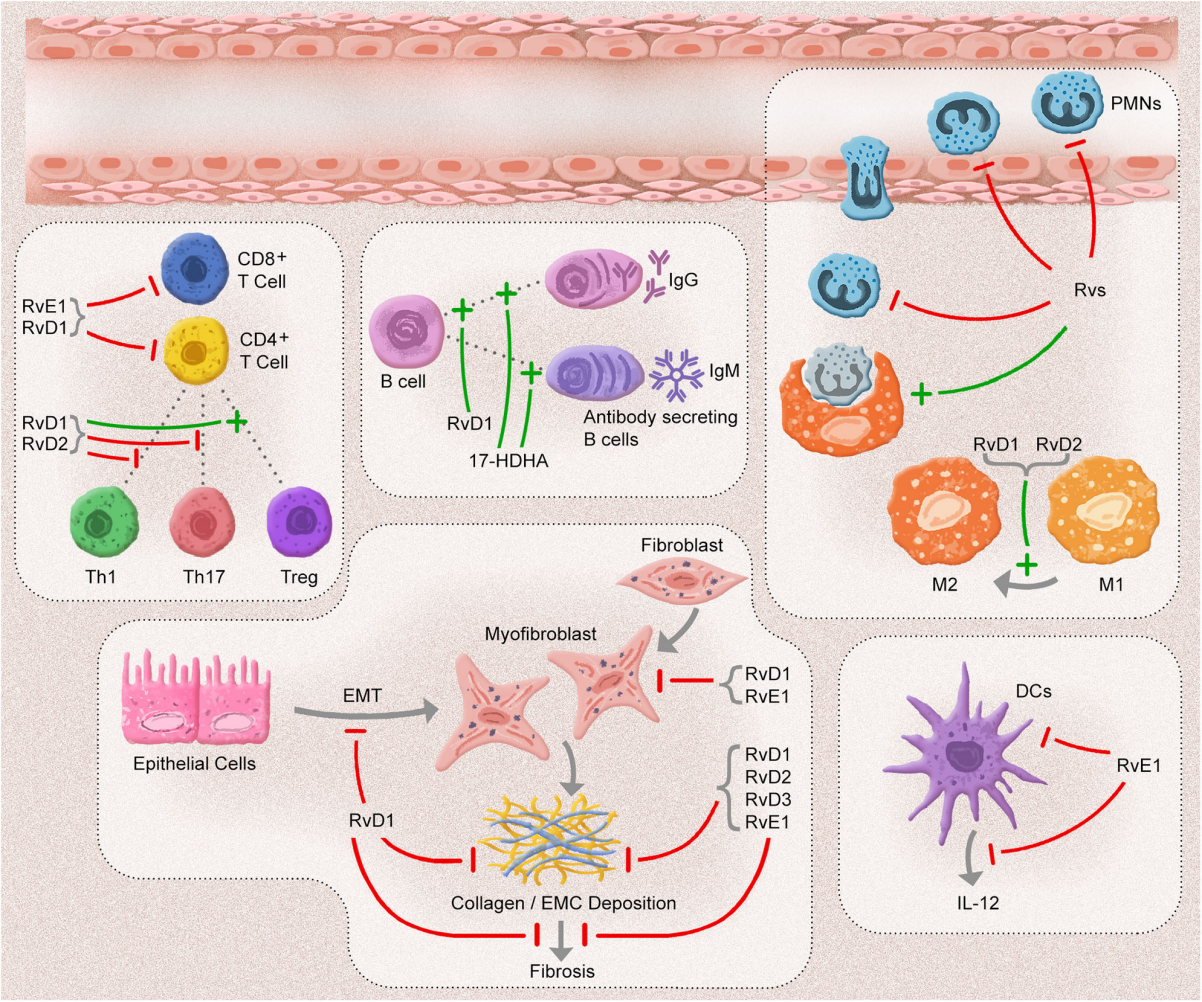
The anti-inflammatory, pro-resolution, and anti-fibrotic effects of Resolvins. In an acute inflammatory response, resolvins (Rvs) inhibit the adhesion, migration, and infiltration of polymorphonuclear leukocytes (PMNs) and enhance the efferocytosis capacity of macrophages. D-series Rvs (RvD1 and RvD2) induce the polarization of macrophages toward to phenotype M2. One of the E-series Rvs, RvE1 blocks the migration and production of interleukin (IL)-12 in dendritic cells (DCs) and the infiltration of CD8^+^ and CD4^+^ cells. RvD1 and RvD2 suppress the inflammatory responses of CD8^+^ T, T helper (Th)1, and Th17 cells, in addition to limiting the differentiation of CD4^+^ T cells into T helper (Th)1 and Th17 cells and promoting the conversion of T regulatory (Treg) cells. 17-hydroxydosahexaenoic acid (17-HDHA) and RvD1 enhance the antibody secretion of B cells. After an inflammatory response, most of Rvs block the development of fibrosis by decreasing collagen deposition and myofibroblast infiltration, as well as by inhibiting epithelial-mesenchymal cell transition (EMT).

The resolution of inflammation is vital for ensuring tissue homeostasis. Any defects in the resolution phase could lead to a prolonged inflammatory response, including increasing PMNs, exaggerated proinflammatory mediator production, increase in the number of apoptotic cells, and inappropriate activation of adaptive immune cells. This unresolved inflammation results in fibrosis of the affected tissue. After the identification of SPMs, many investigators have focused on the effects of SPM on the resolution of inflammation. Rvs are efficacious anti-inflammatory and pro-resolving mediators that play various roles in innate immunity cells. A myriad of studies has confirmed that they influence adaptive immune cells (Figure 2). This exciting anti-fibrotic effect has been supported by the direct effect of these mediators on the regulation of fibrotic cells and cytokines.

The pathogenesis of SSc is associated with vasculopathy, immune dysregulation, and fibrosis (1). It is well-known that progressive chronic inflammation is a part of the disease, while the connection between the resolution of inflammation and SSc still remains unclear. From this viewpoint, any dysfunction of the well-defined anti-fibrotic, anti-inflammatory, and pro-resolving abilities of Rvs may contribute to the progression of SSc. In the future, an accurate understanding of Rvs in SSc may foster the development of novel treatment strategies (4, 111).

## Author Contributions

AA: substantial contributions to the conception of the work, acquisition, and interpretation of data, drafting the article, revising the manuscript critically, providing approval for publication of the content. FR: acquisition of data, drafting the article, revising the manuscript critically, providing approval for publication of the content. SB-R and NP: revising the manuscript critically, providing approval for publication of the content. AT: drafting the article, revising the manuscript critically, providing approval for publication of the content. MM: drafting the article, revising the manuscript critically, providing approval for publication of the content. AP: substantial contributions to the conception of the work, revising the manuscript critically, providing approval for publication of the content. MM-C: substantial contributions to the conception of the work, revising the manuscript critically, providing approval for publication of the content.

## Conflict of Interest

The authors declare that the research was conducted in the absence of any commercial or financial relationships that could be construed as a potential conflict of interest.
